# An additional challenge for head and neck radiologists: anatomic variants posing a surgical risk – a pictorial review

**DOI:** 10.1186/s13244-019-0794-7

**Published:** 2019-12-02

**Authors:** Davide Farina, Davide Lombardi, Martina Bertuletti, Giovanni Palumbo, Ivan Zorza, Marco Ravanelli

**Affiliations:** 1 Department of Radiology, University of Brescia, P.zzale Spedali Civili 1, 25123 Brescia, Italy; 2 Department of Otolaryngology, University of Brescia, Brescia, Italy

**Keywords:** Head and neck, Anatomic variants, CT, MRI, Surgery

## Abstract

Anatomic variants in the head and neck are quite numerous and occur frequently: a minority of them increase the risk of complications during surgical procedures and may be visualized on cross-sectional images. As some of these complications are potentially fatal, awareness (and accurate reporting) of such variants is a basic responsibility of radiologists, particularly when surgery in the pertinent anatomic area is under consideration.

## Key points

- Anatomic variants in head and neck are numerous and frequently encountered
- Some anatomic variants, if not known, can trigger serious complications during surgery
- Radiologists should inform surgeons about the presence of relevant anatomic variants

## Background

Anatomic variants in the head and neck are quite numerous and occur frequently, particularly in the sinonasal region. Some of them, mainly in the paranasal region, are known to predispose to pathology, whereas most bear little (if any) clinical significance. Only a minority of variants increase the risk of complications and iatrogenic damage during surgical procedures. As some of these complications are fatal, awareness and accurate reporting of such variants is a basic responsibility of radiologists. In this pictorial review, anatomic variants posing a surgical risk will be classified under four main categories: abnormal bone pneumatization, bone dehiscence and asymmetry, anomalous vessel course, and anomalous nerve course.

## Abnormal bone pneumatization

In the paranasal area, the Onodi cell is probably the most alarming variant. This cell is the extension of an ethmoid cell above and/or lateral to the sphenoid sinus; hence, it is also referred to as a sphenoethmoid cell. Given its location, it may have an intimate relationship with the optic nerve canal.

In fact, a more restrictive definition of the Onodi cell includes an optic nerve canal protrusion or dehiscence. During endoscopic sinus surgery, the transgression of the walls of an overlooked Onodi cell may result in irreversible optic nerve injury and/or profuse hemorrhage. The prevalence of this anomaly is variable: Shin et al. [1] found an incidence of ~ 30% with a good correlation between multidetector CT (MDCT) and intraoperative findings. However, literature data on its prevalence are inconsistent, with incidence ranging between 10.9% [2] and 65% [3].

**Fig. 1:**
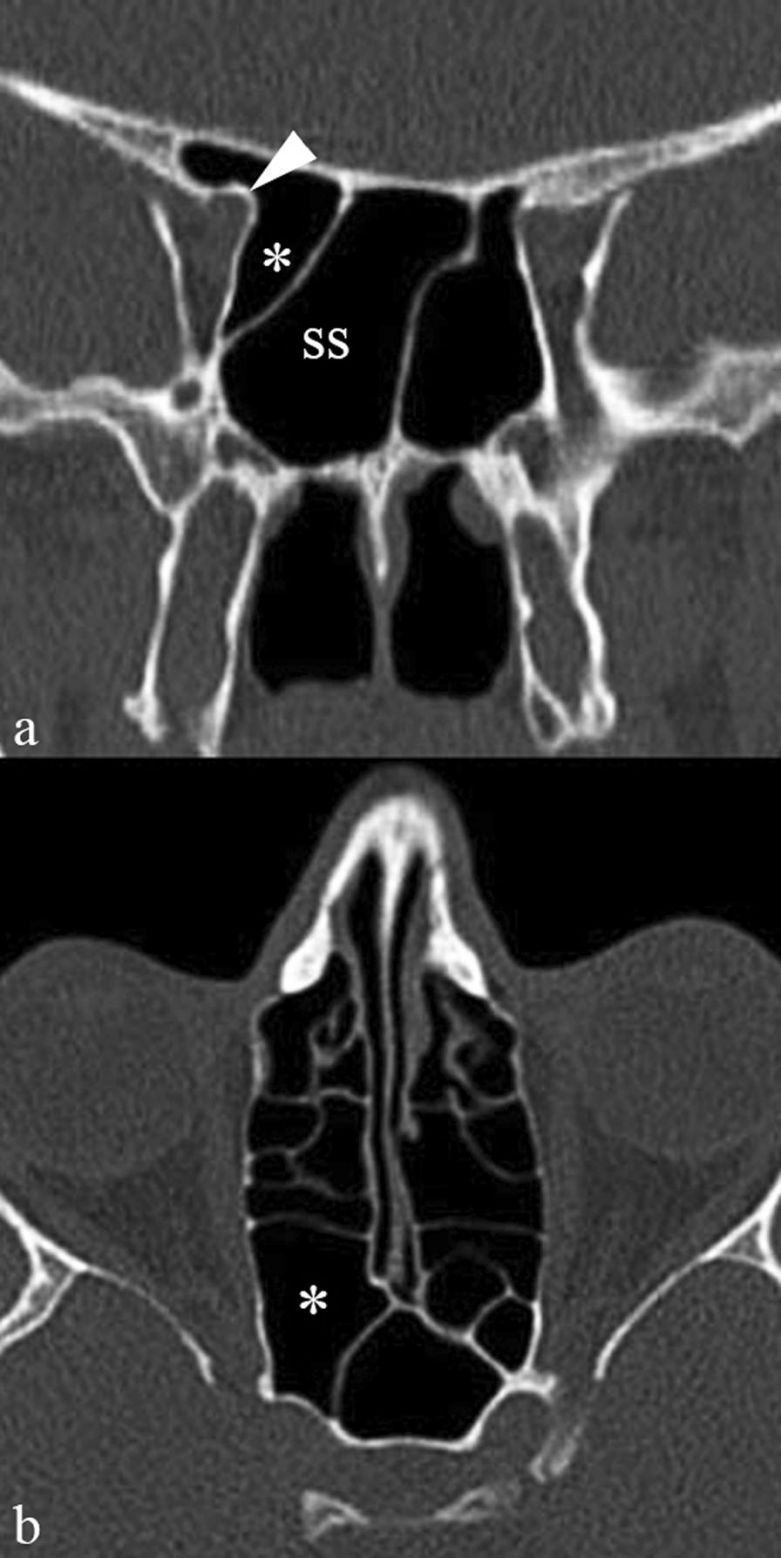
Onodi cell. Coronal (**a**) and axial (**b**) MDCT images show a large air-cell (asterisk) along the superolateral aspect of the right sphenoid sinus (ss), corresponding to an Onodi cell. Pneumatization extends to the anterior clinoid process; consequently, the optic nerve canal protrudes slightly within the Onodi cell (arrowhead)

On cross-sectional scans, the Onodi cell can be best appreciated in coronal plane images, when a horizontal septum is seen crossing the sphenoid sinus lumen (Fig. 1). Then, axial and sagittal reconstructions should be carefully scrutinized to detect the sphenoethmoid recess (the reference point of the actual location of the sphenoid sinus) and confirm the location of the Onodi cell on top of the sphenoid sinus.

Another point of concern for optic nerve injury during surgery is the anterior clinoid process pneumatization. In this case, the risk of iatrogenic damage is related to the thickness of the bony walls of the process and to the degree of pneumatization surrounding the nerve. The prevalence of this variant is low (6–13%) [4].

**Fig. 2:**
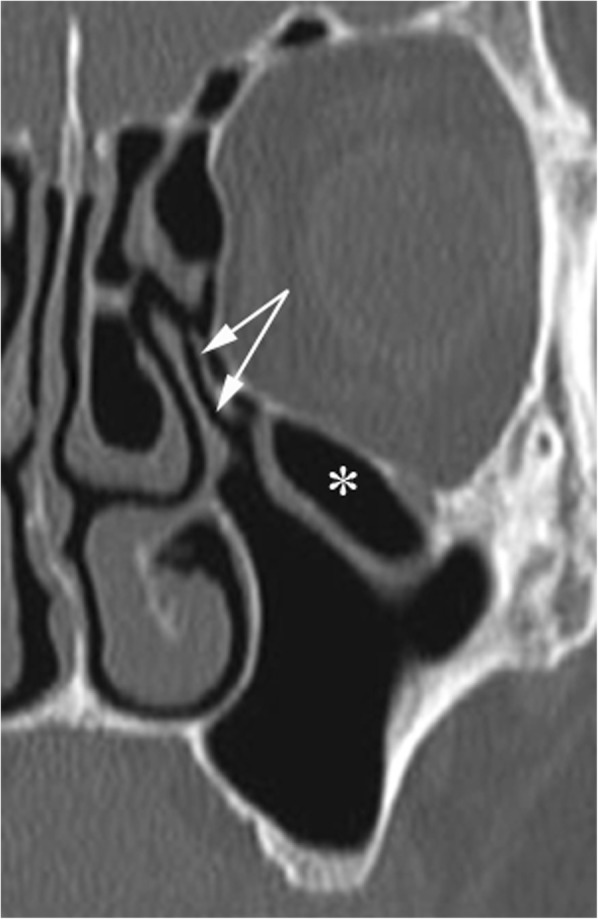
Haller’s cell. Coronal MDCT reformation shows a huge infraorbital cell (asterisk) along the inferior orbital wall, not affecting the caliber of the ethmoid infundibulum (arrows)

The infraorbital (or Haller’s) cell is an extension of ethmoid pneumatization to the orbital wall, inferolateral to the ethmoid bulla (Fig. 2). This cell may obstruct the ethmoid infundibulum, thereby predisposing the patient to maxillary sinusitis and may increase the hazard of orbital penetration during endoscopic sinus surgery [4]; while opening such a cell, in fact, the surgeon may dangerously leverage on the inferomedial orbital wall.

Both clinoid pneumatization and Haller’s cell are readily detected on coronal MDCT scans or on cone beam CT (CBCT) scans.

**Fig. 3:**
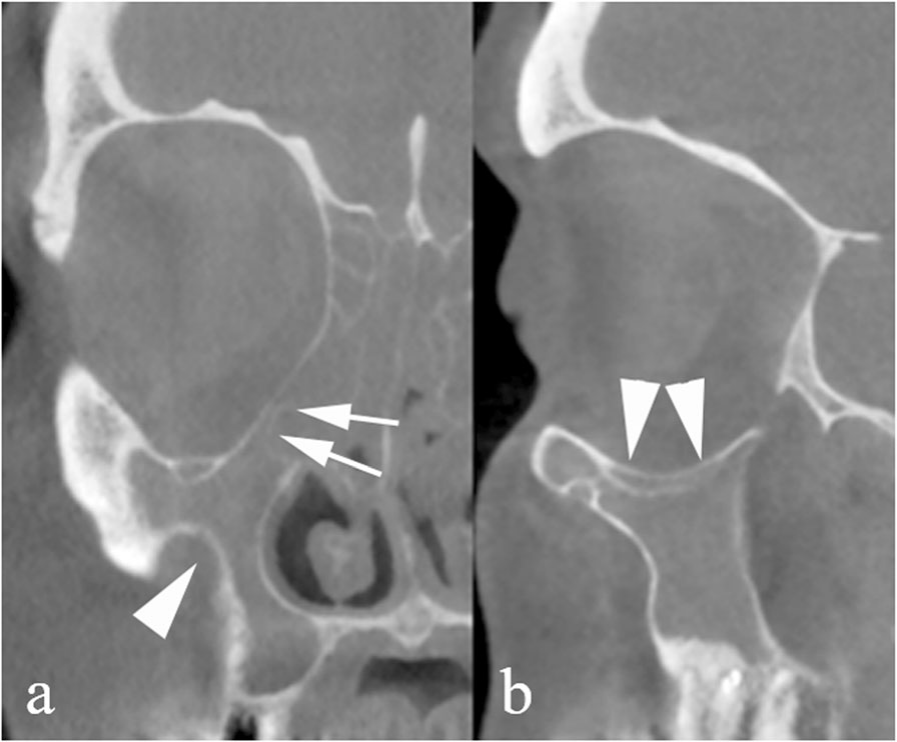
Uncinate process collapse. CBCT reformations on the coronal (**a**) and sagittal (**b**) plane. The right uncinate process (arrows) lies in contact with the inferomedial orbital wall. The maxillary sinus and ethmoid infundibulum are opacified by inflamed mucosa and shrinkage of the lumen, and its walls (arrowheads) are remodeled and display an abnormally concave shape. The condition is referred to as silent sinus syndrome

A unilaterally shrunk maxillary sinus may indicate the collapse of the uncinate process along the inferomedial orbital wall. This anomalous position of the uncinate process enhances the risk of accidental orbital penetration during uncinectomy, a frequent procedure in the early stages of endoscopic sinus surgery. The pathophysiology in this anatomic variant is triggered by adhesion between the uncinate process and the orbital wall; in fact, this induces chronic hypoventilation and negative pressure within the maxillary sinus resulting in shrinkage of the lumen and inflammatory thickening of the mucosa. Negative sinus pressure also induces partial collapse of the orbital floor and increased vertical diameter of the orbit. All of these signs are referred to as silent sinus syndrome [5] and can be easily detected on (CB) MDCT or magnetic resonance imaging (MRI) scans (Fig. 3).

The anatomy of the frontal sinus is largely conditioned by the degree of pneumatization of surrounding air cells (mostly agger nasi cells) and by the cranial attachment of the uncinate process [6]. Some configurations (i.e., supraorbital ethmoid air cell and deep olfactory fossa) significantly influence the complexity of the procedure and may increase the relative risk of complications.

## Bone dehiscence and asymmetry

**Fig. 4:**
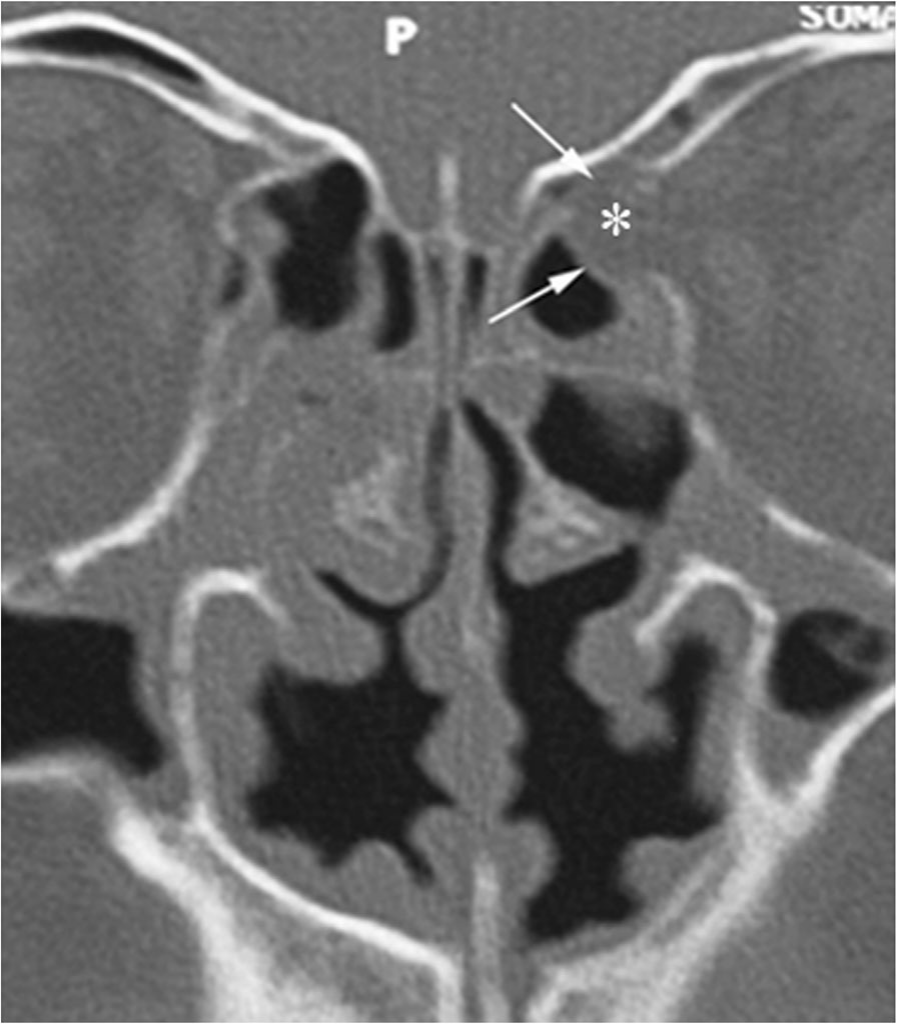
Orbital wall dehiscence. The thin lamina papyracea is focally interrupted on the left side (arrows), where a small amount of extraconal fat tissue (asterisk) protrudes into the ethmoid labyrinth

Focal dehiscence of the orbital wall may occur as a result of trauma; when no history of trauma is reported by the patient, it is a matter of debate whether such an anomaly should be classified as congenital or secondary to minor, clinically overlooked, traumatic events [7]. The risk of iatrogenic orbital injury during endoscopic sinus surgery is quite obvious. (Fig. 4). On MDCT scans displayed with bone-windowing, herniation of the orbital content through gaps in the medial or inferior wall may be easily concealed when the ethmoid or maxillary mucosa is thickened; therefore, whenever gaps are seen on scans with bone windowing, soft tissue reconstructions should be obtained.

**Fig. 5:**
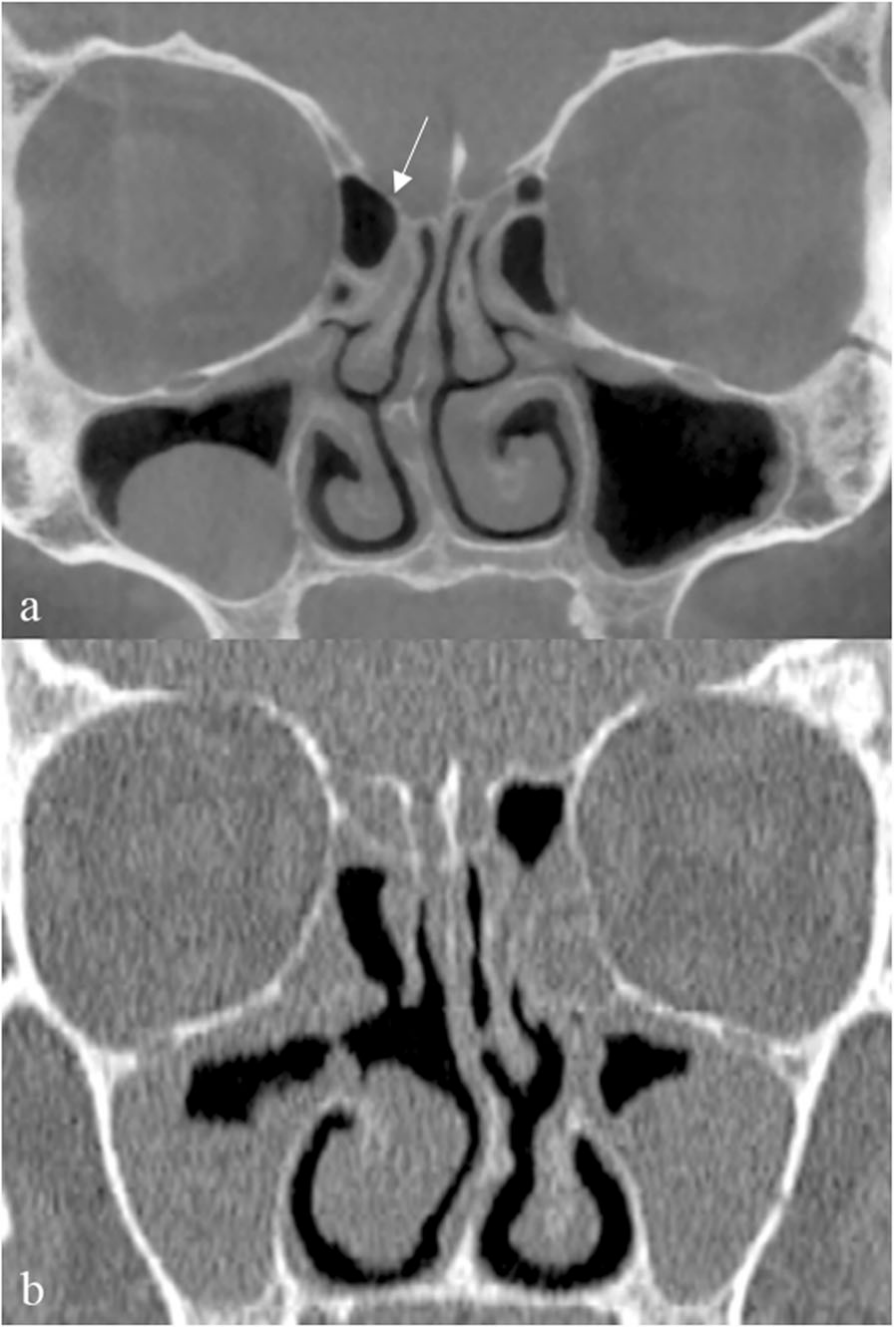
Anatomic variants of the ethmoid roof. CBCT (**a**) shows clear asymmetry, the slope of the right vertical lamella (arrow) being more pronounced than on the contralateral side. **b** MDCT shows a deep olfactory fossa (type III according to the Keros classification)

The anatomic configuration of the ethmoid roof is quite variable. The depth of the cribriform plate is a key point for endoscopic sinus surgery planning; Keros classified it in three groups, according to the length of the vertical lamella of the ethmoid: type I indicates less than 3 mm depth, type III more than 7 mm, type II ranging from 3 mm to 7 mm [8] (Fig. 5). Pre-operative assessment of the depth of the olfactory fossa is crucial because, during surgery, the forces applied to the concha media may lead to breakage of the vertical lamella and to cerebrospinal fluid leak. The Keros type III configuration bears an increased risk of such complication [9, 10]. Meyers and Valvassori [11] proposed a more practical classification: a horizontal line is drawn connecting the cribriform plate to the lateral orbital wall and the depth is defined based on whether the line crosses the upper third of the orbit, the midline, or further below. Furthermore, asymmetry between the two sides, not an infrequent condition, portends an increased surgical risk and should therefore be accurately reported.

**Fig. 6:**
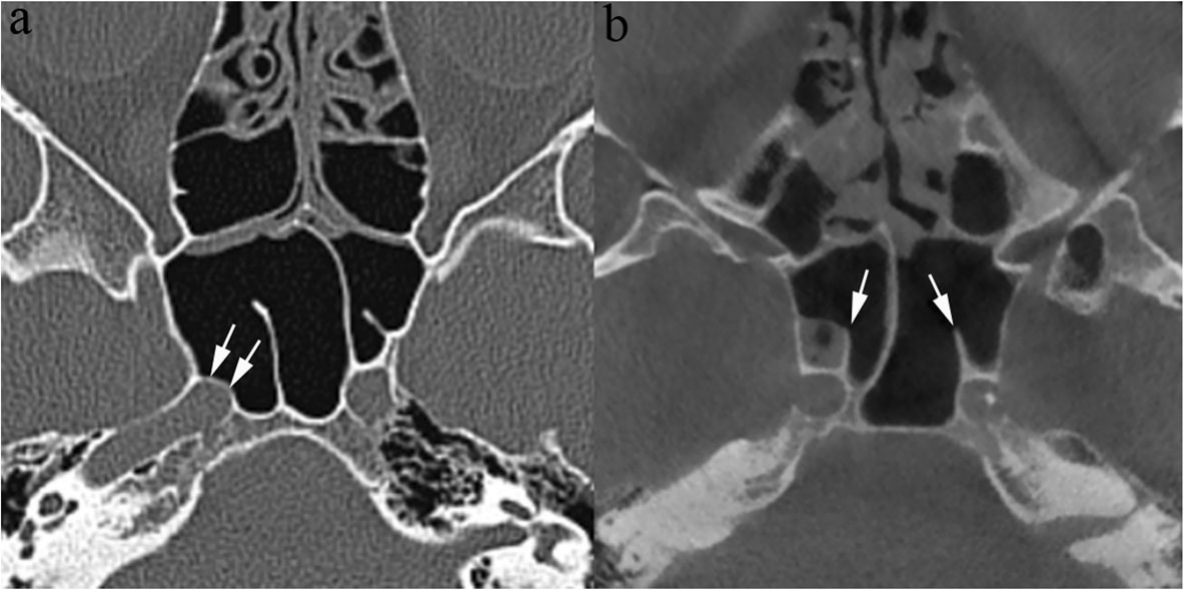
Carotid canal variants. **a** Both canals protrude into the sphenoid sinus cavities; the bony wall is thinned on the right side (arrows). **b** Incomplete bony septa stem from the bony walls of the canals (arrows): during endoscopic surgery, such septa may act as a leverage and facilitate injury of the ICA

When endoscopic surgery of the sphenoid sinus is planned, intraluminal protrusion or focal dehiscence of the vertical tract of the internal carotid artery (ICA) canal increases the risk of surgical injury (Fig. 6). Moreover, bone septa may act as leverage on the carotid canal.

**Fig. 7:**
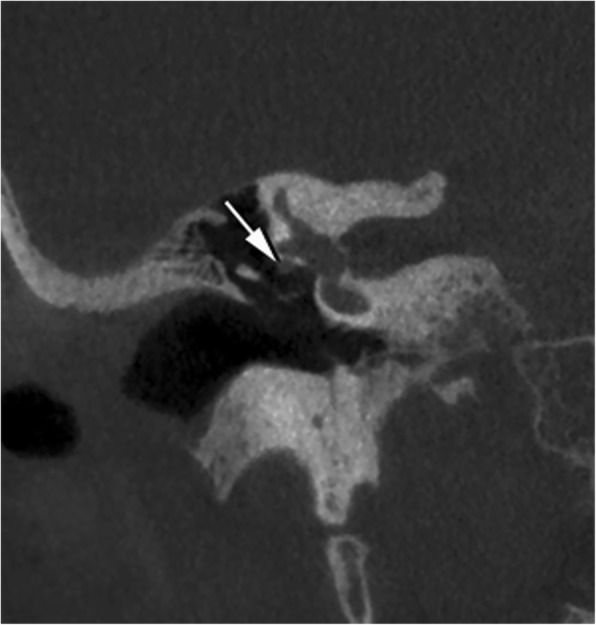
Facial nerve canal dehiscence. Coronal CBCT reconstruction shows dehiscence of the canal and minimal protrusion of the facial nerve (arrow) within the middle ear cavity

In the temporal bone, the tympanic segment of the facial nerve may protrude into the middle ear cavity through a dehiscent bony canal. This is better appreciated on coronal reformations, showing the nerve hanging in the middle ear cavity, strictly contiguous to the oval window (Fig. 7); however, the variant may be obscured by middle ear disease. Bone dehiscence of the facial nerve canal may also be at the second genu; in a review of 202 patients treated surgically for chronic ear disease, dehiscence of the facial nerve canal (overall seen in 8.9%) was equally frequent in the tympanic segment and second genu [12].

**Fig. 8:**
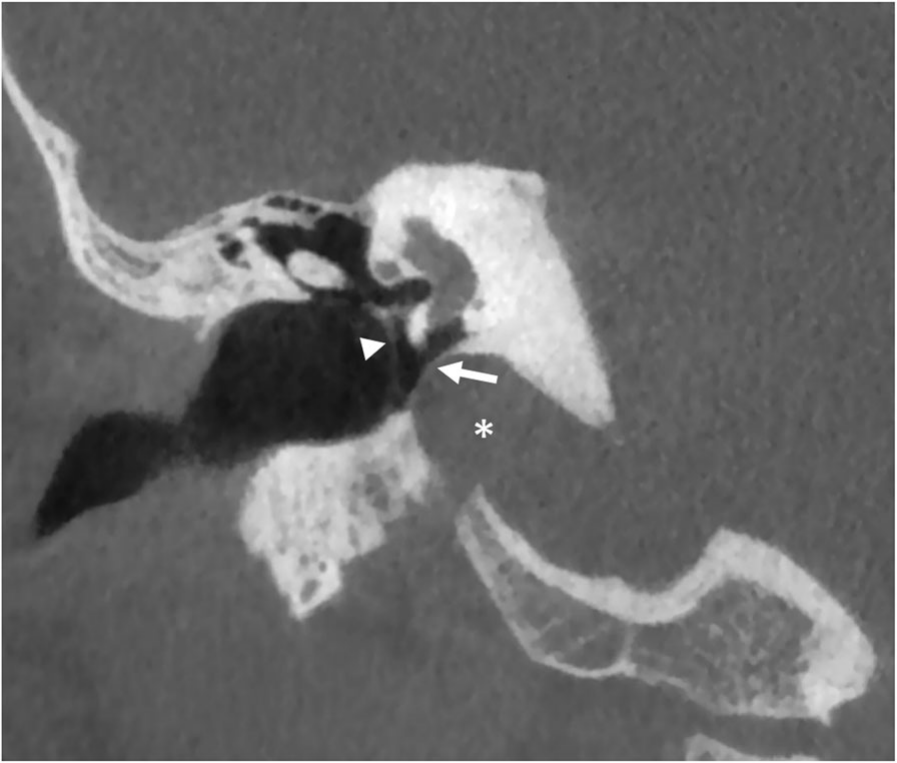
Jugular bulb protrusion*.* Coronal CBCT scan shows prominence of the right jugular bulb (asterisk) into the hypotympanic cavity; the bony plate covering the vein is thinned (arrow). Tympanic membrane (arrowhead)

In addition, the sigmoid plate covering the jugular bulb may be dehiscent; large gaps will result in protrusion of the vein in the mesotympanum, often manifesting with tinnitus and a vascular tympanic membrane (Fig. 8). Occasionally, multiple tegmen defects (honeycomb tegmen) [13] may increase the risk of middle cranial fossa penetration during middle ear surgery.

## Anomalous vessel course

**Fig. 9:**
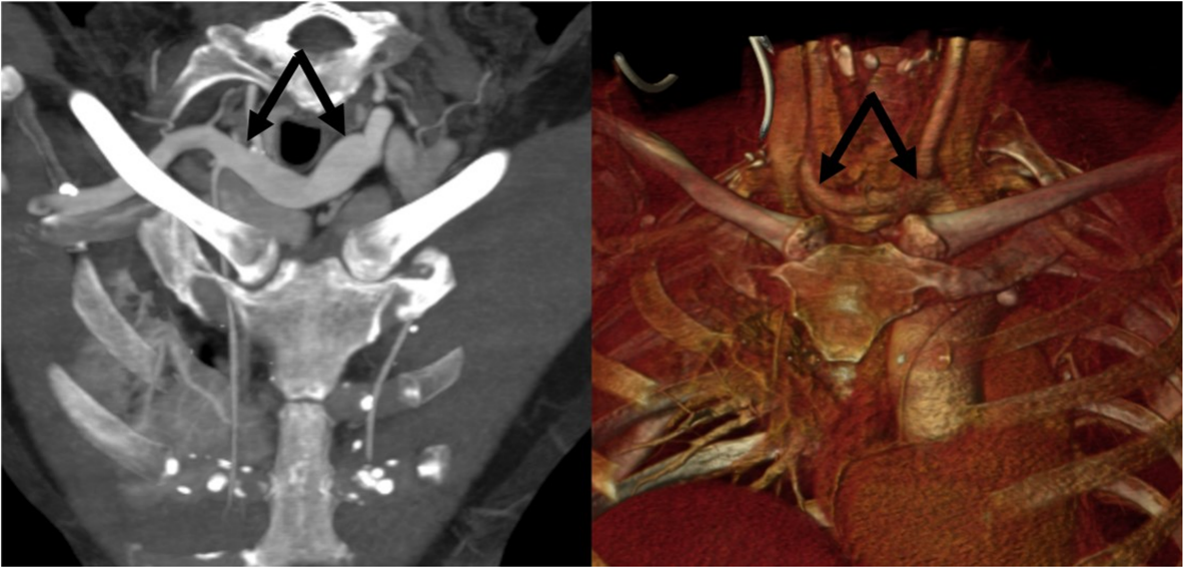
High-riding truncus brachiocephalicus. MIP (**a**) and 3D volume rendering (**b**) reconstruction in an oblique frontal orientation depicting a high-riding truncus brachiocephalicus (arrows) in the lower neck coursing transverse in front of the trachea, high above the sternal manubrium

The high-riding truncus brachiocephalicus is upwardly shifted in the lower neck, with the bifurcation lying close to the thyroid gland. This variant probably develops as a consequence of anomalous regression of the IV arch [14] (Fig. 9).

**Fig. 10:**
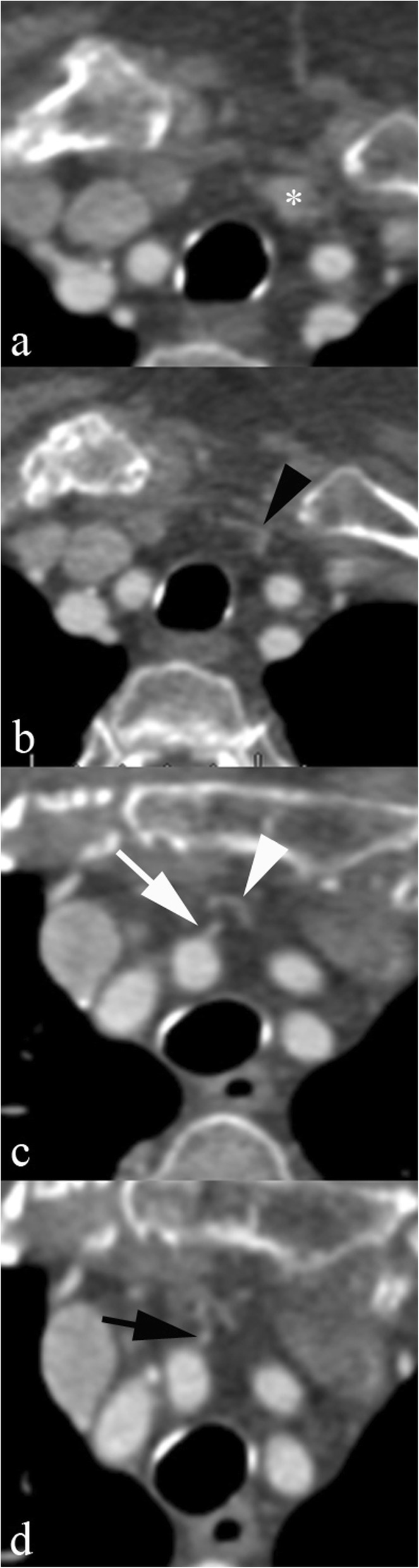
Thyroid ima artery. Axial CT scan: a thin artery arises from the right common carotid artery (arrow in **c**), bends inferiorly (arrow in **d**) and then turns upwards (arrowheads in **b** and **c**) to reach the lower pole of the left thyroid gland lobe (asterisk in **a**)

The caudal part of the thyroid gland can be supplied by a thyroid ima artery, a variant often associated with the absence of inferior thyroid arteries. A thyroid ima artery may arise from the aortic arch, the truncus brachiocephalicus, right common carotid artery, or internal thoracic artery and reaches the thyroid bed coursing along the anterior surface of the trachea [15] (Fig. 10).

Both of these variant vessels may pose a surgical threat, principally when tracheostomy or thyroid/parathyroid surgery is planned; identification on cross-sectional imaging requires careful assessment of vascular structures in the peritracheal soft tissues.

**Fig. 11:**
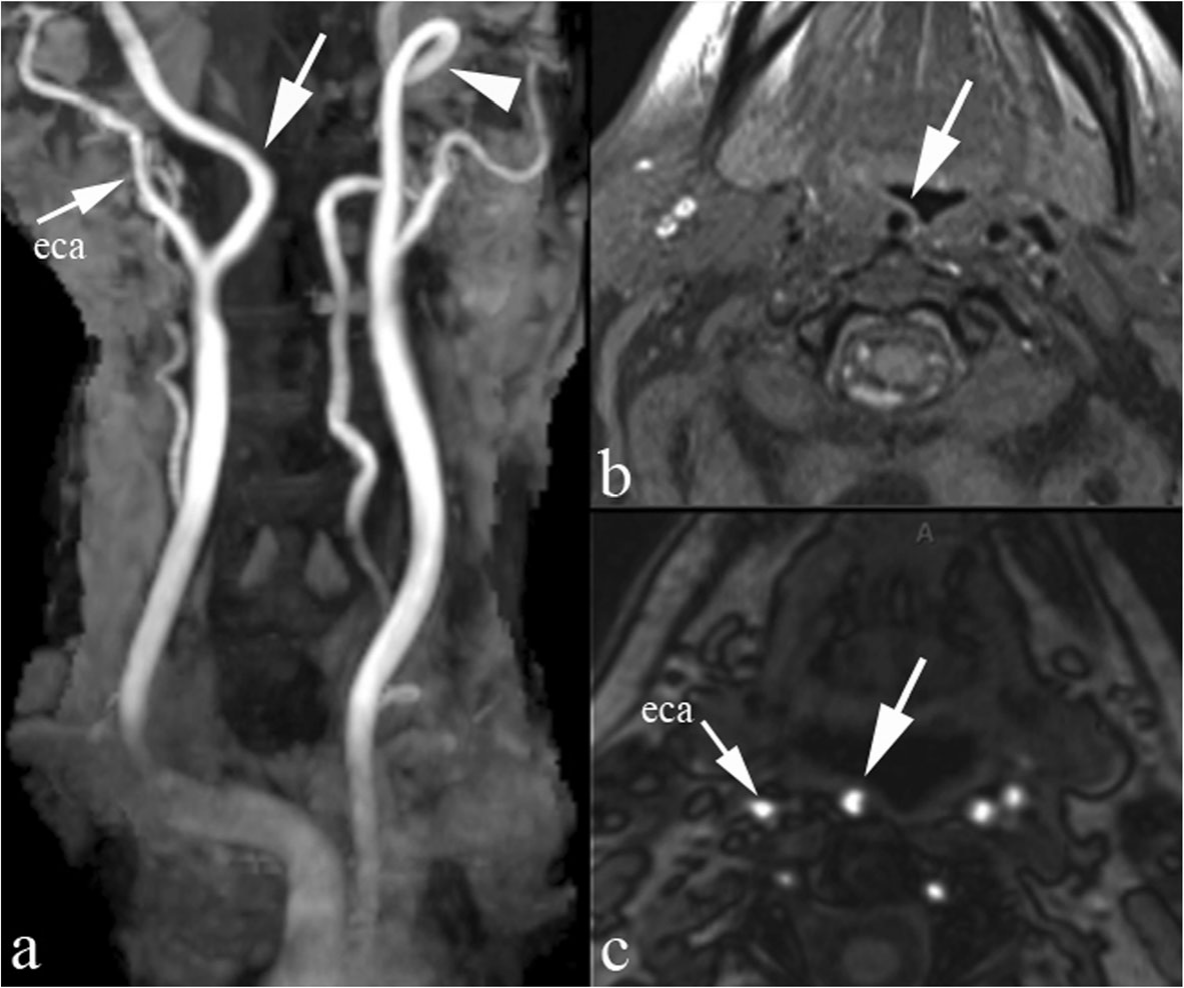
Retropharyngeal carotid artery*.* MRI 3D TOF, thick maximum intensity projection (MIP) reconstruction on coronal plane (**a**) and native axial partition (**c**); fat suppressed axial SE T1 with contrast administration (**b**). The right ICA (large arrows) bends medially reaching the midline behind the posterior oropharyngeal wall. Coiling of the left ICA (arrowhead). eca external carotid artery

A retropharyngeal carotid artery relates to the relatively common medial shift of the ICA (less frequently the common or external carotid artery); it can be bilateral, a condition referred to as “kissing carotids” and is prevalently seen at the oropharyngeal and hypopharyngeal level [16] (Fig. 11).

The incidence of this variant is linearly related to patient age; thus, it is possibly explained by increased tortuosity and atherosclerotic changes or by hypertension. Severe complications may be generated by retropharyngeal carotid artery injury, even during routine surgery such as tonsillectomy or peritonsillar abscess drainage. Interestingly, a change in position (from and to retropharyngeal) on MDCT examinations acquired at different time points has been described in 6.3% of cases [17].

**Fig. 12:**
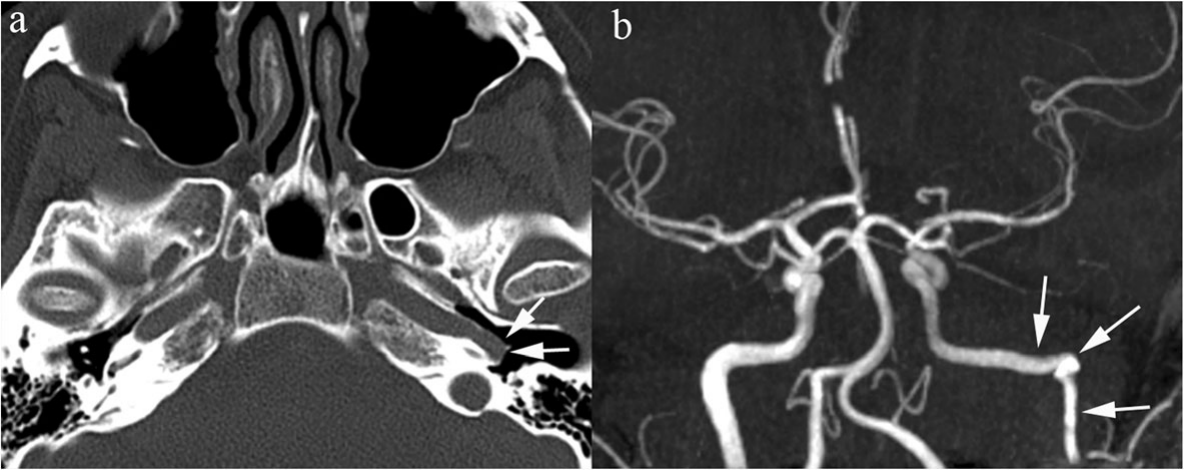
Aberrant ICA. Axial MDCT scan (**a**) shows asymmetry of petrous ICA, the left coursing on the surface of the cochlear promontory (arrows). **b** MIP reconstruction in the coronal plane of a TOF sequence shows pinched contour of the ICA (arrows)

In the temporal bone, the vertical portion of the petrous internal carotid artery may be undeveloped and bypassed by hypertrophied inferior tympanic and caroticotympanic arteries, coursing in the hypotympanum: this condition is named an aberrant ICA [18] (Fig. 12). It may manifest as pulsatile tinnitus and mimic a vascular mass on otoscopy or may remain asymptomatic. CBCT/MDCT may indicate the absence of the vertical portion of the ICA, presence of a hypotympanic soft tissue mass, enlargement of the inferior canaliculus, and absence of bone coverage on the intratympanic segment of the vessel. As the hypotympanic soft tissue mass may be obscured by diffuse inflammatory opacification of the middle ear, awareness of this condition is crucial. On MRI, time-of-flight (TOF) angiography shows a pinched contour at the intersection of the vertical and horizontal segments of the ICA.

**Fig. 13:**
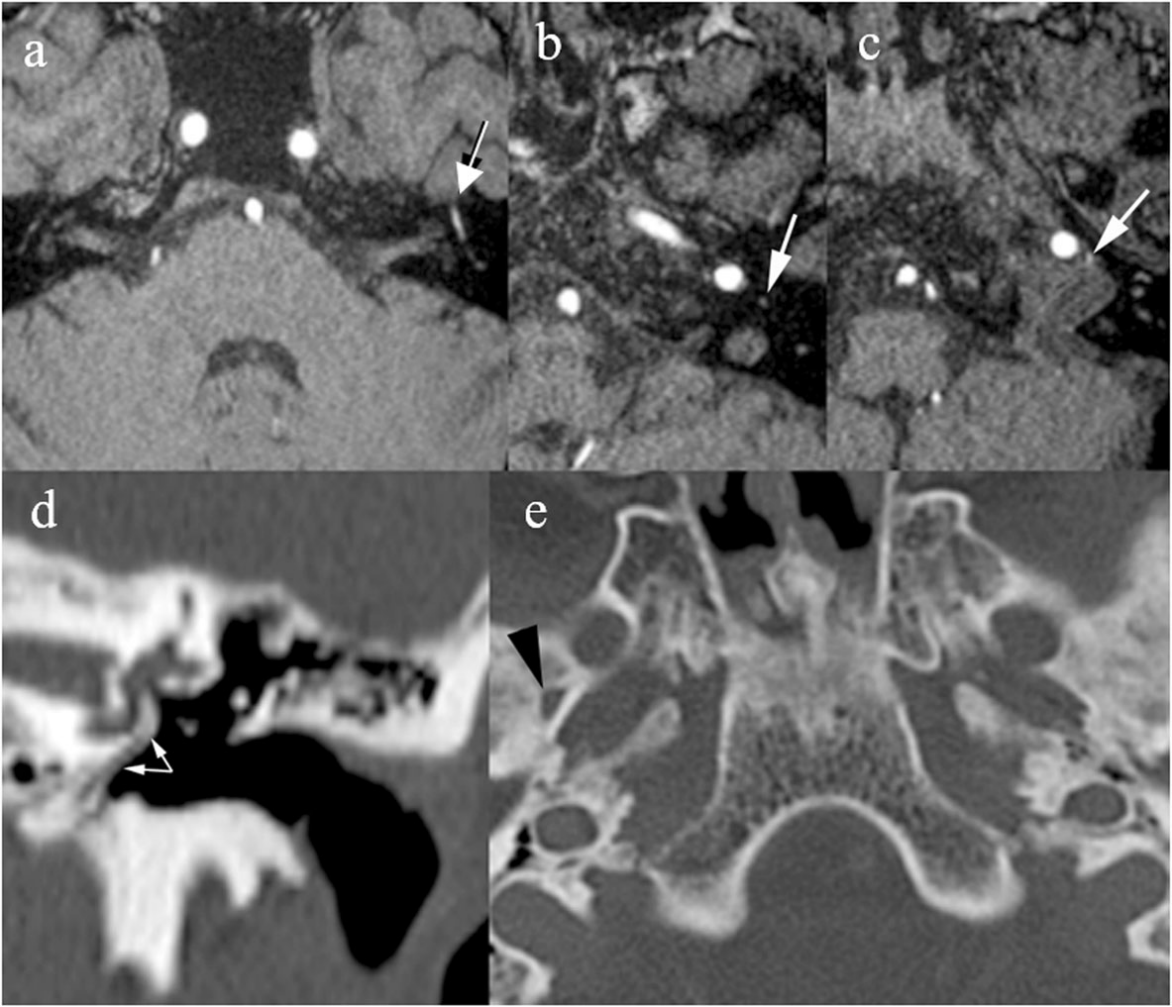
Persistent stapedial artery. Axial partitions, TOF sequence with selective venous flow suppression (**a–c**). A thin arterial structure (arrows) is seen close to the ICA (**c**) along the promontory (**b**) and along the tympanic segment of the facial nerve (**a**). CT of the brain (**d,e**): coronal multiplanar reformation (MPR) and native axial acquisition. A linear structure runs along the cochlear promontory (arrows), and the left foramen spinosum is absent (black arrowhead points to the right foramen, normally present). Findings are consistent with a persistent stapedial artery

Rarely, hemorrhage during middle ear surgery may be produced by injury to a persistent stapedial artery. During fetal life, the stapedial artery provides a connection between branches of the external and internal carotid artery; in about 0.05% of cases, the vessel does not regress and may be seen in its entire course, arising from the petrous ICA [19]. The persistent stapedial artery crosses the antero-medial hypotympanum, courses between the crura of the stapes to reach the facial nerve canal, and follows retrogradely a short segment of its tympanic portion up to the geniculate ganglion, where it enters the extradural space in the middle cranial fossa [20]. When the stapedial artery persists, the middle meningeal artery arises from it, and thus MDCT and MRI images show the absence of the foramen spinosum; in addition, a small vascular canal may be seen along the cochlear promontory and the facial nerve canal will have an abnormally large diameter. High-resolution submillimetric (i.e., 0.9 mm or less isotropic voxel) MRI sequences with gadolinium will show the vessel, along with an abnormal enhancement along the second segment of the petrous facial nerve (Fig. 13).

## Anomalous nerve course

In the sinonasal area, anomalous nerve course is often the consequence of abnormal pneumatization and/or dehiscence of the bony walls of their canals. As a result, cranial nerves in the maxillofacial area and temporal bone may be seen coursing within air cavities. The maxillary and vidian nerve may protrude into the sphenoid sinus when the pterygoid root is pneumatized thus creating a lateral recess. Less commonly, the infraorbital nerve may protrude into or hang in the maxillary sinus.

**Fig. 14:**
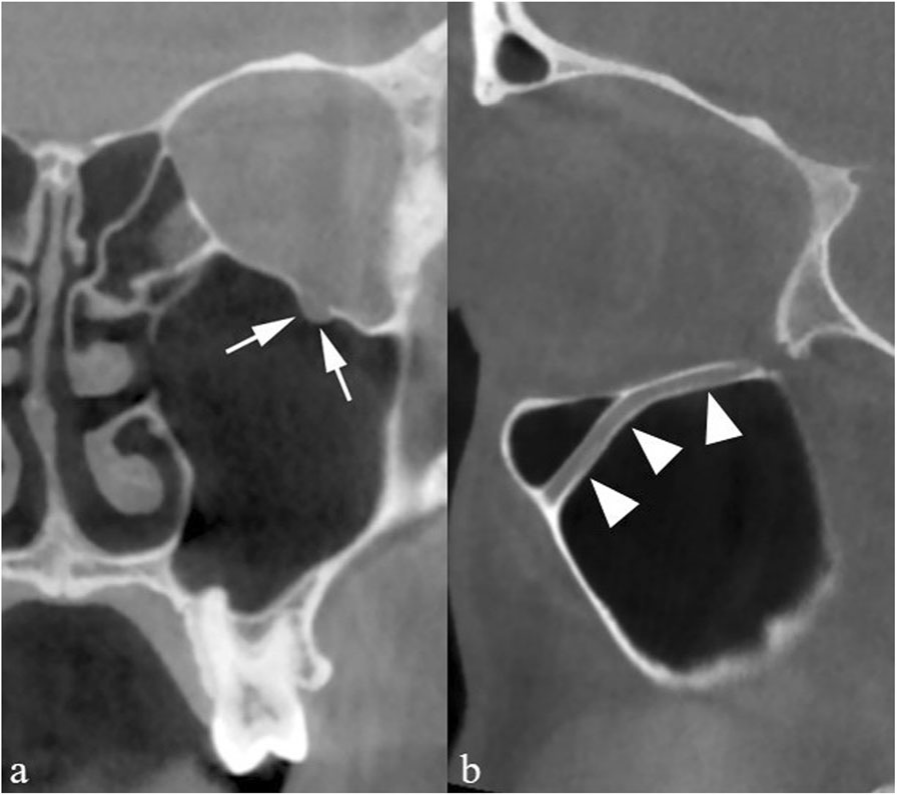
Anatomical variant of nerve course. Coronal CBCT reformation (**a**) showing dehiscence of the bony walls of the infraorbital canal (arrows) and minor protrusion of the nerve within the maxillary sinus; sagittal CBCT reformation (**b**) showing the nerve hanging in the maxillary sinus cavity (arrowheads), covered by intact nerve canal walls

Surgical risk is amplified if the bony laminae surrounding such nerves are dehiscent (Fig. 14); an intrasinusal infraorbital nerve is at risk during endoscopic surgery when hidden by or coursing within the laminae of an infraorbital (Haller’s) cell [21].

**Fig. 15:**
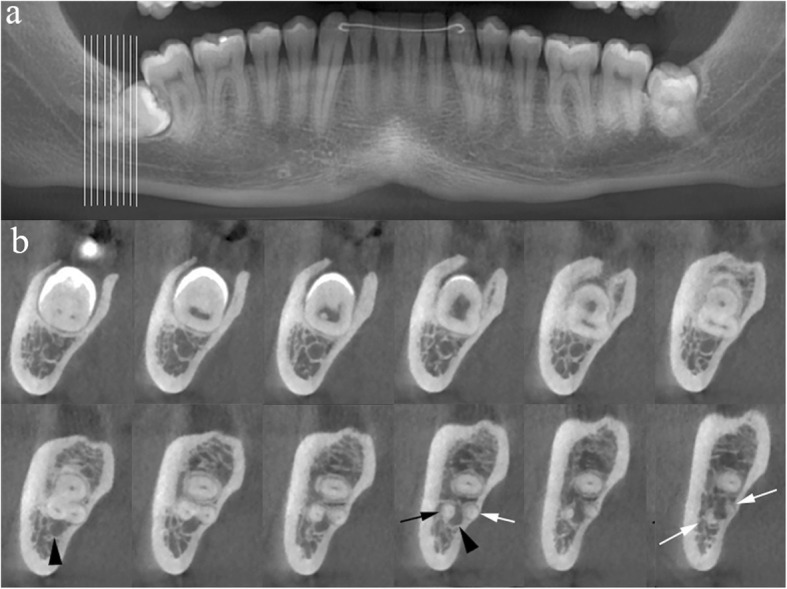
Anatomical variant of nerve course. CBCT of the mandible, panorex reconstruction (**a**) indicating the range of the cross-sectional images in **b**. The roots (arrows) of the included right third molar encompass and remodel the mandibular canal (arrowheads), increasing the risk of nerve damage during tooth extraction

Two nerve course variants in the mandible demand extreme caution during tooth extraction: the mandibular nerve canal may be completely encircled by molar teeth roots (Fig. 15); additionally, a retromolar canal is seen in ~ 25% of cases branching from the most proximal part of the mandibular canal to reach the retromolar fossa: nerves supplying molar teeth may be damaged during extraction of included elements. Both conditions are exquisitely demonstrated by CBCT images [22]. A retrospective review in 136 patients with 257 impacted third molars identified several risk factors for iatrogenic nerve damage: contact between tooth roots and nerve, absent cortication of the nerve canal, nerve shape (teardrop and dumb-bell shape), and nerve position relative to dental roots (lingual and interradicular) [23].

In the lower neck, relevant nerve course variants may be detected only during surgery, such as the extralaryngeal ramification of the recurrent nerve and the numerous variations of the course of the spinal accessory nerve [24].

**Fig. 16:**
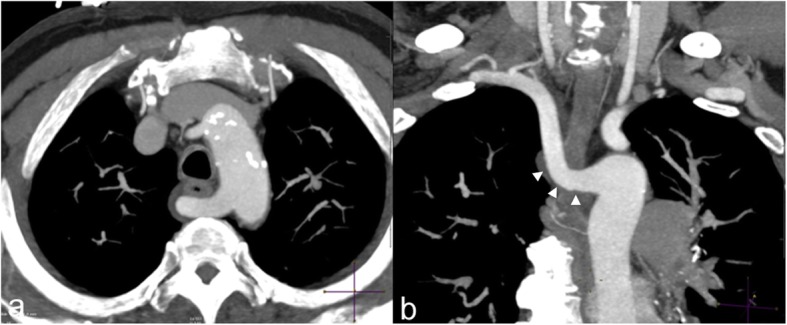
Aberrant right subclavian artery. Axial (**a**) and coronal (**b**) MIP thin reconstruction of a CT angiography of chest showing aberrant retroesophageal course of the right subclavian artery (arrowheads)

Though not directly demonstrable on cross-sectional images, the presence of a non-recurrent right laryngeal nerve may be heralded by an indirect finding, namely the aberrant right subclavian artery (Fig. 16). During embryologic life, the inferior laryngeal nerves supply V–VI branchial arches; on the right side, as these arches disappear, the nerve course retracts cranially being finally “trapped” around the right subclavian artery. Anomalous regression of the right IV arch results in an independent origin of the subclavian artery from the arch and a non-recurrent inferior laryngeal nerve unrestrained by the subclavian artery retracts cranially in the neck [25]. A non-recurrent course of the right laryngeal nerve increases the risk of iatrogenic nerve injury during thyroid and parathyroid surgery.

## Conclusion

Some anatomic variants in the supra- and infrahyoid neck may increase surgical risk and, if overlooked, have the potential to generate serious complications. Often such variants produce subtle findings on cross-sectional images. It is the responsibility of the radiologist reporting a head and neck scan to carefully scrutinize the regional anatomy in search of variants and to report on them, particularly when surgery is under consideration.

## Data Availability

Data sharing is not applicable to this article as no datasets were generated or analyzed during the current study.

## Abbreviations

CBCT: Cone beam CT
CT: Computed tomography
MDCT: Multidetector CT
MRI: Magnetic resonance imaging
